# Supplementary material for: mtR_find: A Parallel Processing Tool to Identify and Annotate RNAs Derived from the Mitochondrial Genome
Source: Int J Mol Sci. 2023 Feb 22;24(5):4373. doi: 10.3390/ijms24054373 (PMC10001721; doi:10.3390/ijms24054373)
Supplement: Supplementary file 1 [file ijms-24-04373-s001.zip › Supplementary File S12.pdf]

Comparing results from simulation and tool:

To check the individual elements of the output generated from simulation and the tool, the CSV files from both the output are loaded as data frame and checked for concordance as described below:

Comment lines are indicated by “#”, command lines are indicated by “>>>” and the results if applicable by [out X]

import python module PANDAS and load CSV files as data frames

>>> import pandas as pd
>>> sim_output = pd.read_csv("lnc_RNA_sim.csv")
>>> tool_output = pd.read_csv("lnc_RNA.csv")
#check if column names are identical in both data frame for comparison. First step printing column names. Checking the number of columns and finding differences

>>> list(sim_output)
[out 1] ['sequence', 'Length', 'type', 'bio-type', 'strand', 'orientation', 'annotation', 'Sequence alignment', 'Sequence start position(bp)', 'Sequence end position(bp)', 'gene-boundary:start(bp)', 'gene-boundary:end(bp)', 'substitutions', 'total_count', 'stimulated-file-1', 'stimulated-file-2', 'stimulated-file-3', 'stimulated-file-4']
>>> list(tool_output)
[out 2] ['sequence', 'Specific-ID', 'Length', 'type', 'bio-type', 'strand', 'orientation', 'annotation', 'Sequence alignment', 'Sequence start position(bp)', 'Sequence end position(bp)', 'gene-boundary:start(bp)', 'gene-boundary:end(bp)', 'substitutions', 'total_count', 'stimulated-file-1', 'stimulated-file-2', 'stimulated-file-3', 'stimulated-file-4']
>>> list(sim_output) == list(tool_output)
[out3] False
>>> len(list(sim_output))
[out 4] 18
>>> len(list(tool_output))
[out 5] 19

the tool output has one additional column. Find that and delete the column. For comparing the contents of two data frame, two dataframes need to have identical column labels and same number of columns

```
>>> list(set(tool_output)-set(sim_output))
```

[out 6] ['Specific-ID']

tool output has an additional column "Specific-ID". Delete it

```
>>> tool_output.drop("Specific-ID", axis = 1, inplace = True)
```

the contents from the tool and the simulation has to be in a specific order for element wise comparison. Both the data frame is sorted based on "sequence" alphabetically. The resulting data frame would not have index in the proper order. For comparing two data frame the index should match as well. So the index is reset for both dataframes.

```
>>> sim_output.sort_values("sequence", ascending = True, inplace =True)
>>> tool_output.sort_values("sequence", ascending = True, inplace =True)
>>> sim_output.sort_values(drop = True, inplace = True)
>>> sim_output = sim_output.fillna(0)
>>> tool_output = tool_output.fillna(0)
>>> difference = (sim_output != tool_output).stack()
>>> diff_df = difference[difference]
>>> diff_df. index.names = ['id', 'col']
>>> diff_df
id      col
0   substitutions    True
4   substitutions    True
5   substitutions    True
14  substitutions    True
dtype: bool
```

```
>>> pd.DataFrame({"value_in_sim": sim_val_change, "value_in tool": tool_val_change}, index = changed.index)
                        value_in tool    value_in_sim
id      col
0       substitutions       -98A0           98A0
4       substitutions       -144C0          144C0
5       substitutions       -88G0           88G0
14      substitutions       -74T0           74T0
```

There are only 4 entries that show a difference and the difference is with "-" in front of the elements in tool output that was done for parsing information.
