# Supplementary material for: mtR_find: A Parallel Processing Tool to Identify and Annotate RNAs Derived from the Mitochondrial Genome
Source: Int J Mol Sci. 2023 Feb 22;24(5):4373. doi: 10.3390/ijms24054373 (PMC10001721; doi:10.3390/ijms24054373)
Supplement: Supplementary file 1 [file ijms-24-04373-s001.zip › Supplementary File S14.pdf]

Comment lines are indicated by “#”, command lines are indicated by “>>>” and the results if applicable by [out X]

# extracting sequences from MINTbase

```
>>> import pandas as pd
>>> import os
>>> min =pd.read_csv("mintbase.txt", sep="\t",skiprows=4)
>>> min_seq = min["Fragment sequence"].tolist()
```

# extracting DE sequences from dataset1

```
>>> files = [ f for f in os.listdir(os.getcwd()) if f.endswith(".csv")]
>>> files
[out 1] ['hepcvsno-cancer2_annotated.csv', 'hepbvsno-cancer2_annotated.csv',
'hepcvsuninfected2_annotated.csv', 'hepbvsuninfected2_annotated.csv']
>>> seq = []
>>> for f in files:
...     seq.extend(pd.read_csv(f)["sequence"].tolist())
...
>>> len(seq)
[out 2] 1598
>>> seq = list(set(seq))
>>> len(seq)
[out 3] 1017
>>> master =pd.read_csv("../mt-sRNA_count.csv")
>>> len(master)
[out 4] 2120
>>> diff = master.loc[master["sequence"].isin(seq)]
>>> len(diff)
[out 5] 1017
```

#extract only mtsRNAs mapping to mitochondrial tRNA genes

```
>>> tRNA = diff.loc[diff["bio-type"] =="Mt_tRNA"]
```

```
>>> len(tRNA)
[out 6] 783
```

#checking how many DE tRfs identified in dataset-1 is not in MINTbase

```
>>> novel = tRNA.loc[~tRNA ["sequence"].isin(min_seq)]
>>> len(novel)
[out 7] 365
```

#checking how many mtsRNAs have sequence start site or end site outside mitochondrial tRNA gene boundary

```
>>> list(novel)
[out 8] ['sequence', 'Specific-ID', 'General-ID', 'subtype', 'type', 'bio-type', 'strand', 'orientation', 'annotation', 'Sequence alignment', 'Sequence start position(bp)', 'Sequence end position(bp)', 'gene-boundary:start(bp)', 'gene-boundary:end(bp)', 'substitutions', 'total_count', 'SRR1274016', 'SRR1274008', 'SRR1274003', 'SRR1274017', 'SRR1274004', 'SRR1273998', 'SRR1274001', 'SRR1274006', 'SRR1274013', 'SRR1274012', 'SRR1274015', 'SRR1274000', 'SRR1274005', 'SRR1274007', 'SRR1274010', 'SRR1274009', 'SRR1274011', 'SRR1274014', 'SRR1274002', 'SRR1273999']
>>> novel.insert(1,"end_shift","")
>>> novel.insert(1,"start_shift","")
>>> novel["start_shift"] = novel["Sequence start position(bp)"]-novel["gene-boundary:start(bp)"].astype(int)
>>> novel["end_shift"] = novel["Sequence end position(bp)"]-novel["gene-boundary:end(bp)"].astype(int)
>>> len(novel.loc[novel["start_shift"].isin(range(-3,4,1))])
[out 9] 174
>>> len(novel.loc[novel["end_shift"].isin(range(-3,4,1))])
[out 10] 92
>>> novel.to_csv("Additional_file11.csv", sep=",",index=False)
>>> novel2 = novel.loc[(novel["start_shift"].isin(range(-3,4,1)))|(novel["end_shift"].isin(range(-3,4,1)))]
>>> novel3 = novel2.loc[~((novel2["start_shift"]==0)|(novel2["end_shift"]==0))]
```

```
>>> len(novel3)
[out 11] 42
```

# checking how many tRfs that are not present in MINTbase have rpm greater than one in atleast one library

```
>>> total =pd.read_csv("../sRNA_count.csv")
>>> list(total)
[out 12] ['read', 'total_count', 'SRR1274016', 'SRR1274008', 'SRR1274003', 'SRR1274017',
'SRR1274004', 'SRR1273998', 'SRR1274001', 'SRR1274006', 'SRR1274013', 'SRR1274012',
'SRR1274015', 'SRR1274000', 'SRR1274005', 'SRR1274007', 'SRR1274010', 'SRR1274009',
'SRR1274011', 'SRR1274014', 'SRR1274002', 'SRR1273999']
>>> list(total)[2:]
[out 13] ['SRR1274016', 'SRR1274008', 'SRR1274003', 'SRR1274017', 'SRR1274004', 'SRR1273998',
'SRR1274001', 'SRR1274006', 'SRR1274013', 'SRR1274012', 'SRR1274015', 'SRR1274000',
'SRR1274005', 'SRR1274007', 'SRR1274010', 'SRR1274009', 'SRR1274011', 'SRR1274014',
'SRR1274002', 'SRR1273999']

>>> for i in list(total)[2:]:
...     print ("processing column " + str(i))
...     col_sum = total[i].sum()
...     total[i] =  total[i].apply(lambda x: (float(x/col_sum)*1000000))
...
[out 14]
processing column SRR1274016
processing column SRR1274008
processing column SRR1274003
processing column SRR1274017
processing column SRR1274004
processing column SRR1273998
processing column SRR1274001
processing column SRR1274006
processing column SRR1274013
```

```
processing column SRR1274012
processing column SRR1274015
processing column SRR1274000
processing column SRR1274005
processing column SRR1274007
processing column SRR1274010
processing column SRR1274009
processing column SRR1274011
processing column SRR1274014
processing column SRR1274002
processing column SRR1273999
>>> test = novel["sequence"].tolist()
>>> filtered =  total.loc[total["read"].isin(test)]
>>> len(filtered)
[out 15] 365
>>> filtered2 = filtered[list(filtered)[2:]]
>>> len(filtered2[(filtered2 >= 1).any(axis=1)])
[out 16] 365
```

#checking the count of mtsRNAs not in MINTbase by annotation and other characteristics

```
>>> from collections import Counter
>>> Counter(novel["annotation"].tolist())
[out 17] Counter({'Ser2': 43, 'Met': 42, 'Glu': 39, 'Val': 38, 'Phe': 31, 'Gln': 26, 'Lys': 25, 'Tyr': 22,
'Leu2': 21, 'Leu': 17, 'Ala': 11, 'Thr': 10, 'His': 9, 'Arg': 7, 'Asp': 7, 'Gly': 4, 'Ile': 4, 'Asn': 4, 'Ser1': 3,
'Trp': 2})
>>> Counter(novel["subtype"].tolist())
[out 18] Counter({'tRF-5': 214, 'i-tRF-5': 56, 'i-tRF-3': 43, 'tRF-3': 42, 'tRNA-half-5': 8, 'tRNA-half-3':
2})
>>> Counter(novel["type"].tolist())
[out 19] Counter({'normal_1_mismatch': 356, 'CCA_0_mismatch': 9})
>>> Counter(novel["bio-type"].tolist())
[out 20] Counter({'Mt_tRNA': 365})
```

```
>>> Counter(novel["strand"].tolist())
[out 21] Counter({'H': 291, 'L': 74})
```
