# Supplementary material for: mtR_find: A Parallel Processing Tool to Identify and Annotate RNAs Derived from the Mitochondrial Genome
Source: Int J Mol Sci. 2023 Feb 22;24(5):4373. doi: 10.3390/ijms24054373 (PMC10001721; doi:10.3390/ijms24054373)

Supplementary File S8, Table S1

Comparison		Count number from simulation	Count number obtained from mt_R find
**Filename**	stimulated-file-1	25165	25165
	stimulated-file-2	26740	26740
	stimulated-file-3	26328	26328
	stimulated-file-4	28058	28058
	**Total count**	**106291**	**106291**
**Bio type**	mt_rRNA	**7182**	**7182**
	mt_tRNA	71568	71568
	Non protein-coding	7252	7252
	Protein coding	20289	20289
	**Total count**	**106291**	**106291**

Table S2

Mitochondrial gene	Start position	End position	Strand
**tRNA-Phe**	577	647	Heavy
**mtSSU rRNA**	648	1601	Heavy
**tRNA-Val**	1602	1670	Heavy
**mtLSU rRNA**	1671	3229	Heavy
**tRNA-Leu**	3230	3304	Heavy
**ND1**	3307	4262	Heavy
**tRNA-Ile**	4263	4331	Heavy
**tRNA-Gln**	4329	4400	Light
**tRNA-Met**	4402	4469	Heavy
**ND2**	4470	5511	Heavy
**tRNA-Trp**	5512	5579	Heavy
**tRNA-Ala**	5587	5655	Light
**tRNA-Asn**	5657	5729	Light
**tRNA-Cys**	5761	5826	Light
**tRNA-Tyr**	5826	5891	Light
**COI**	5904	7445	Heavy
**tRNA-Ser1**	7446	7514	Light
**tRNA-Asp**	7518	7585	Heavy
**COII**	7586	8269	Heavy
**tRNA-Lys**	8295	8364	Heavy
**ATP8**	8366	8572	Heavy
**ATP6**	8527	9207	Heavy
**COIII**	9207	9990	Heavy
**tRNA-Gly**	9991	10058	Heavy
**ND3**	10059	10404	Heavy
**tRNA-Arg**	10405	10469	Heavy
**ND4L**	10470	10766	Heavy

**ND4**	10760	12137	Heavy
**tRNA-His**	12138	12206	Heavy
**tRNA-Ser2**	12207	12265	Heavy
**tRNA-Leu2**	12266	12336	Heavy
**ND5**	12337	14148	Heavy
**ND6**	14149	14673	Light
**tRNA-Glu**	14674	14742	Light
**CytB**	14747	15887	Heavy
**tRNA-Thr**	15888	15953	Heavy
**tRNA-Pro**	15956	16023	Light

Table S3

Gene	Acronym	Type	Criteria
	tRF-1	tRF-1	Mt-sRNAs mapping to 3’ end of rRNA or protein-coding genes preceding a mitochondrial tRNA gene.
	tRF-2	tRNA-half-5’	1) The sequence is longer than or equal to a half of the total gene length, and the sequence spans the 5’ half of a tRNA gene. 2) Sequence start position is ± 3 nt from the tRNA gene start position.
	tRF-4	tRNA-half-3’	1) The sequence is longer than or equal to a half of the total gene length, and the sequence spans the 3’ half of a tRNA gene. 2) Sequence end position is ± 3 nt from the tRNA gene end position.
**tRNA**	tRF-5	tRF-5’	1) The sequence is shorter than a half of the total gene length. 2) Sequence start position is ± 3 nt from the tRNA gene start position.
	tRF-3	tRF-3’	1) The sequence is shorter than a half of the total gene length. 2) Sequence end position is ± 3 nt from the tRNA gene end position.
	tRF-6	i-tRF-5	1) The sequence is shorter than a half of the total gene length. 2) Sequence start position is within 5’ half of tRNA, and it is not within a range ± 3 nt from the tRNA gene start position.
	tRF-7	i-tRF-3	1) The sequence is shorter than a half of the total gene length. 2) Sequence start position is within 3’ half of tRNA, and its end

			position is not within a range ± 3 nt from the tRNA gene end position.
	5H	5prime-half	1) Sequence length is greater than or equal to half the length of total gene length and sequence spans the 5’ half of gene boundary. 2) Sequence start position is ± 3 from the gene end position.
	3H	3prime-half	1) Sequence length is greater than or equal to half the length of total gene length and sequence spans the 3’ half of gene boundary 2) Sequence end position is ± 3 from the gene start position.
**protein coding/rRNA**	5p	5prime	Sequence end position is ± 3 from the gene end position.
	3p	3prime	Sequence start position is ± 3 from the gene start position.
	i-5p	i-5prime	Sequence end position is not in ± 3 from the gene start position and is within 5’ half of gene length.
	i-3p	i-3prime	Sequence start position is within 3’ half of gene and not ± 3 from the gene start position.

Table S4

**Species**	**Abbreviation**
Homo sapiens	hsa
Danio rerio	dre
Mus musculus	mmu
Ratus norvegicus	rno
Xenopus sp.	xen
Gallus gallus	gga

Table S5

Strand	Region	% distribution	Type	sub-distribution	Orientation	Substitutions	Length
**H**	28 genes	15	itRF-5, itRF-3 and randomly fragments	15 %	sense	no	15-35
	tRNA	40	tRNA-half-3	7.5%	sense	no	half the length of mature tRNA
			tRF-1	7.5%	sense	no	16-21
			tRF-5	10 %	sense	yes	18-22
			tRF-3	7.5%	sense	no	15-20
			tRNA-half-5	7.5%	sense	yes	half the length of mature tRNA
	protein-coding	20	random	15	sense	no	15-22
				5	anti-sense	no	22-25
	rRNA	20	random	15	sense	no	15-24
				5	anti-sense	no	18-23
**L**	random location	5	coding	5	sense	no	15-35

Table S6

Total no. of sequences	Length of sequence (range)	Strand	Distribution	Region	Orientation	Substitutions
16	75-150	H	1	non-coding	sense	no
			6	coding	sense	no
			4	coding	anti-sense	yes
		L	5	coding	sense	no

Figure S1

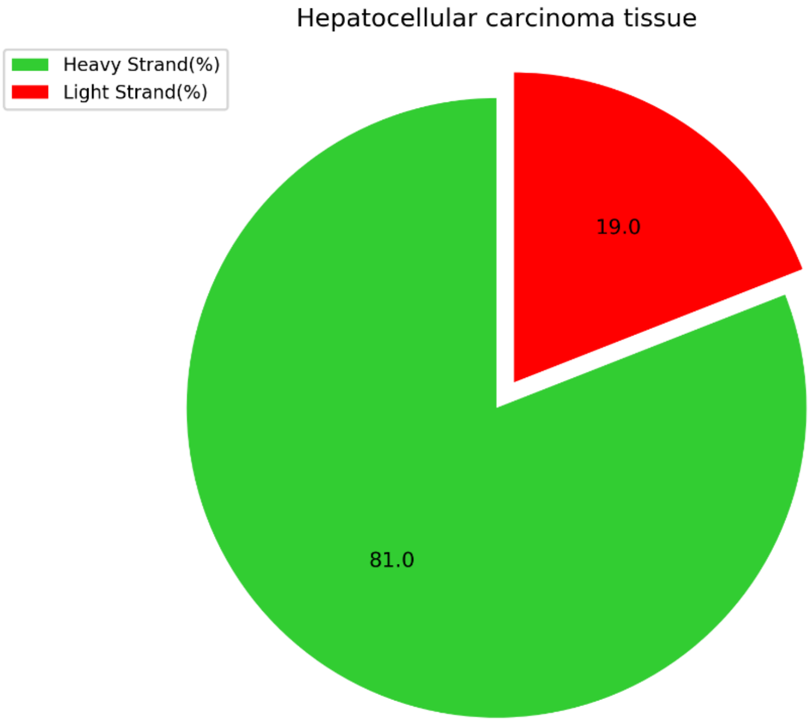
Hepatocellular carcinoma tissue
Heavy Strand(%)
Light Strand(%)
19.0
81.0

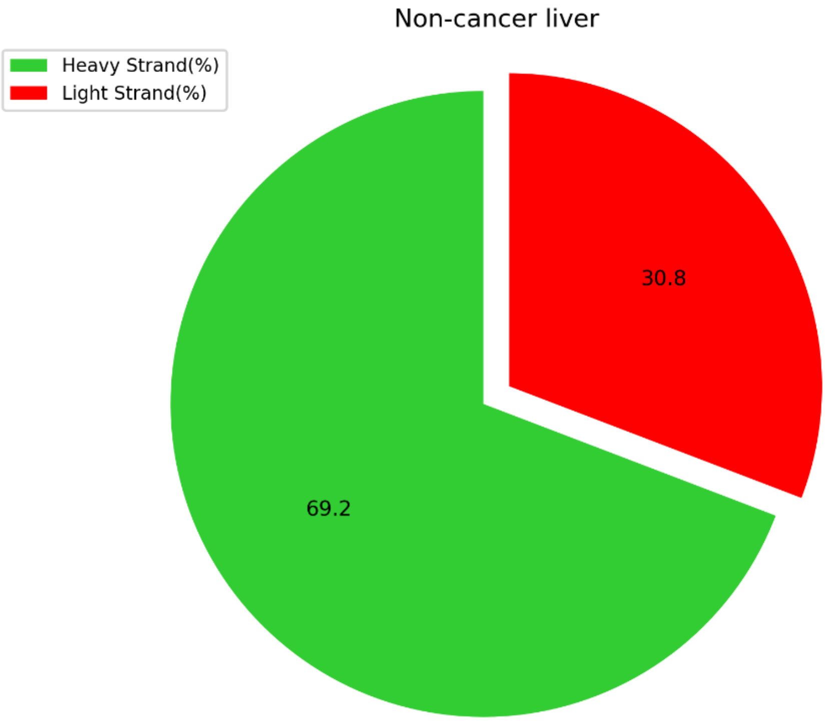
Non-cancer liver
Heavy Strand(%)
Light Strand(%)
30.8
69.2

Figure S2

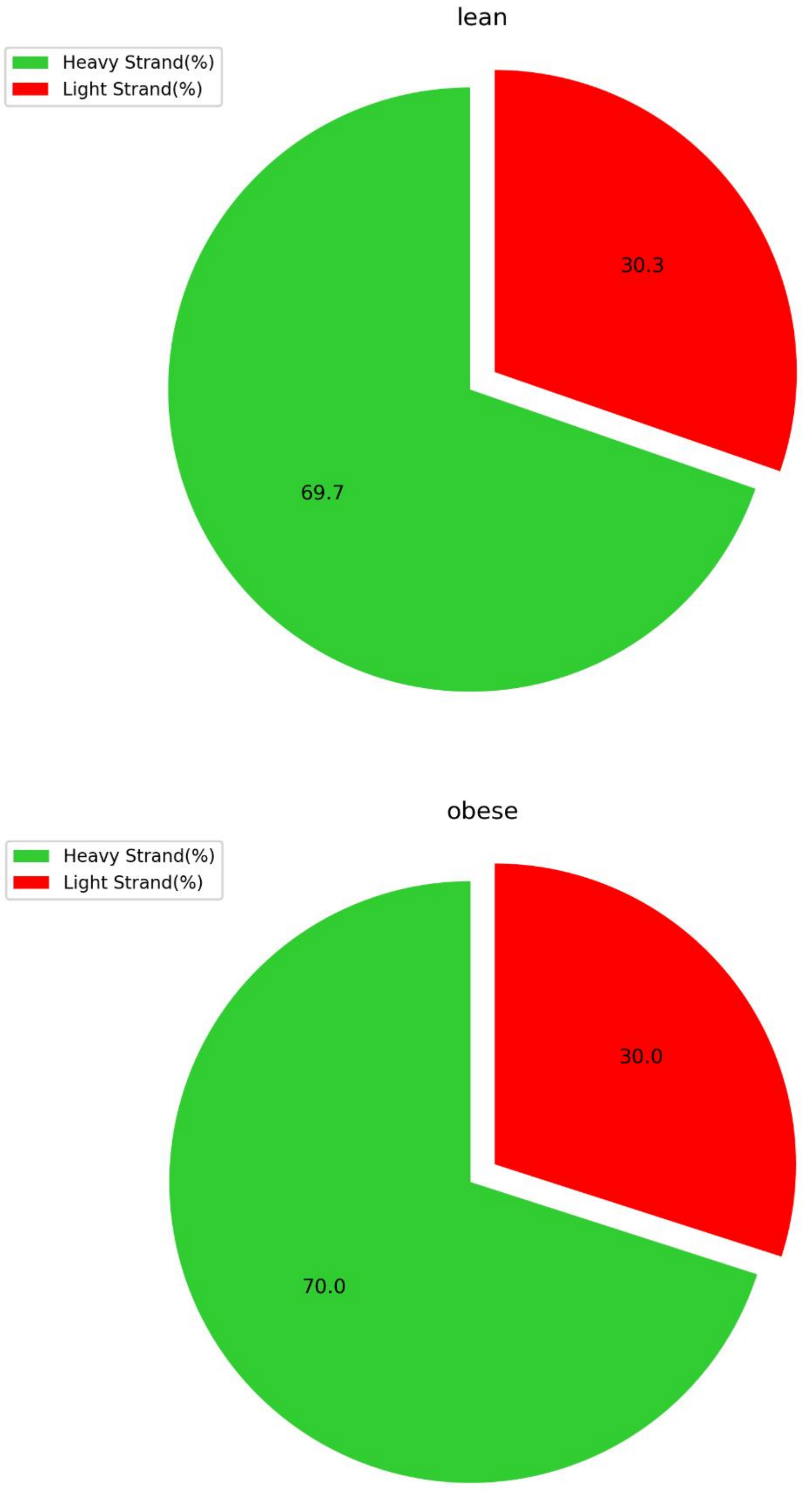
lean
Heavy Strand(%)
Light Strand(%)
30.3
69.7
obese
Heavy Strand(%)
Light Strand(%)
30.0
70.0

Figure S3

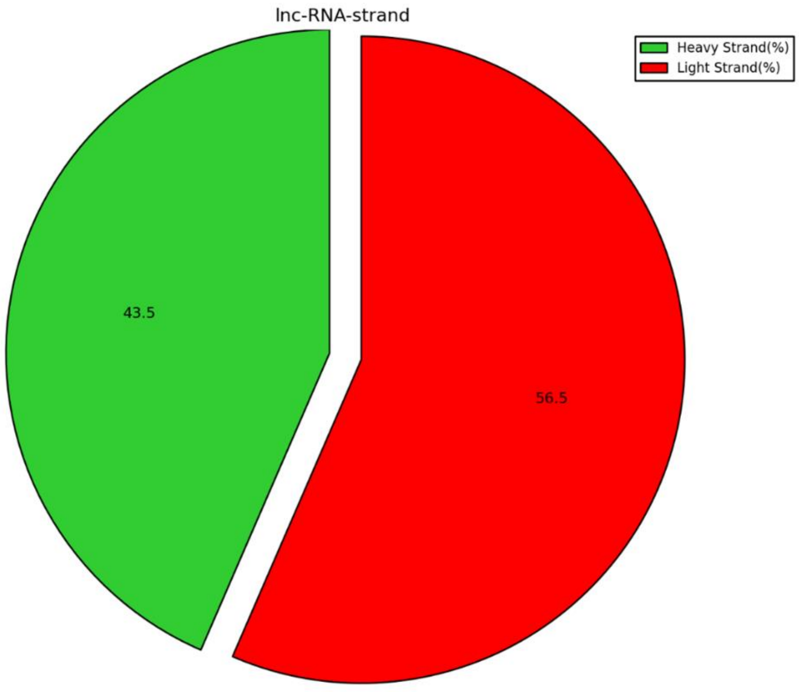

Figure S4A

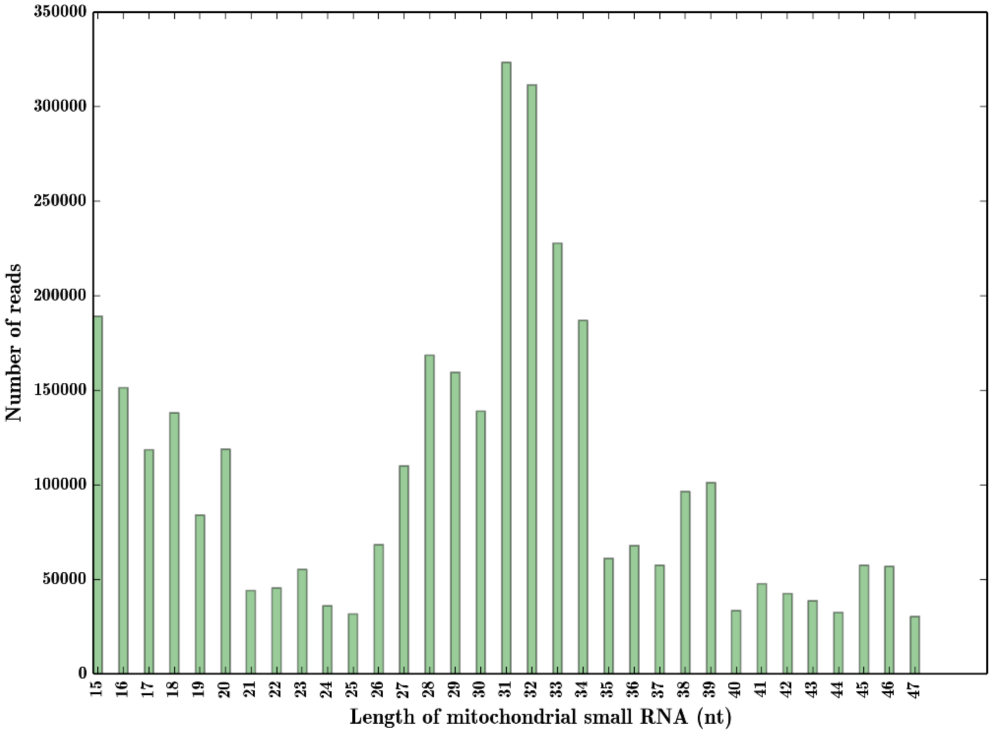

Figure S4B

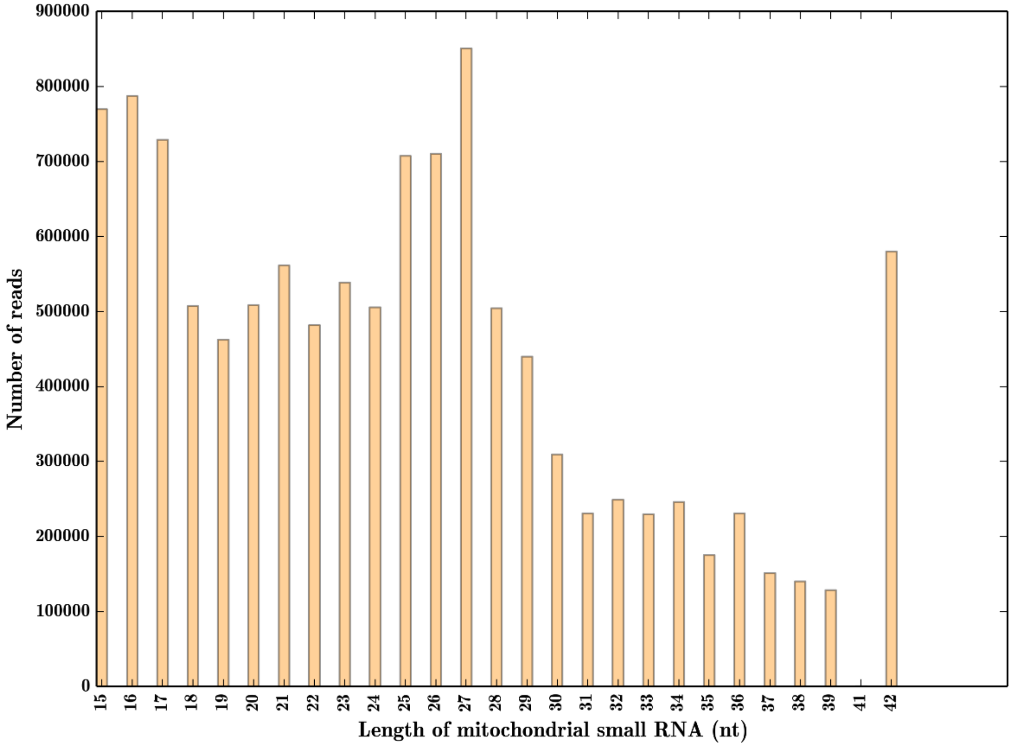

Figure S4C

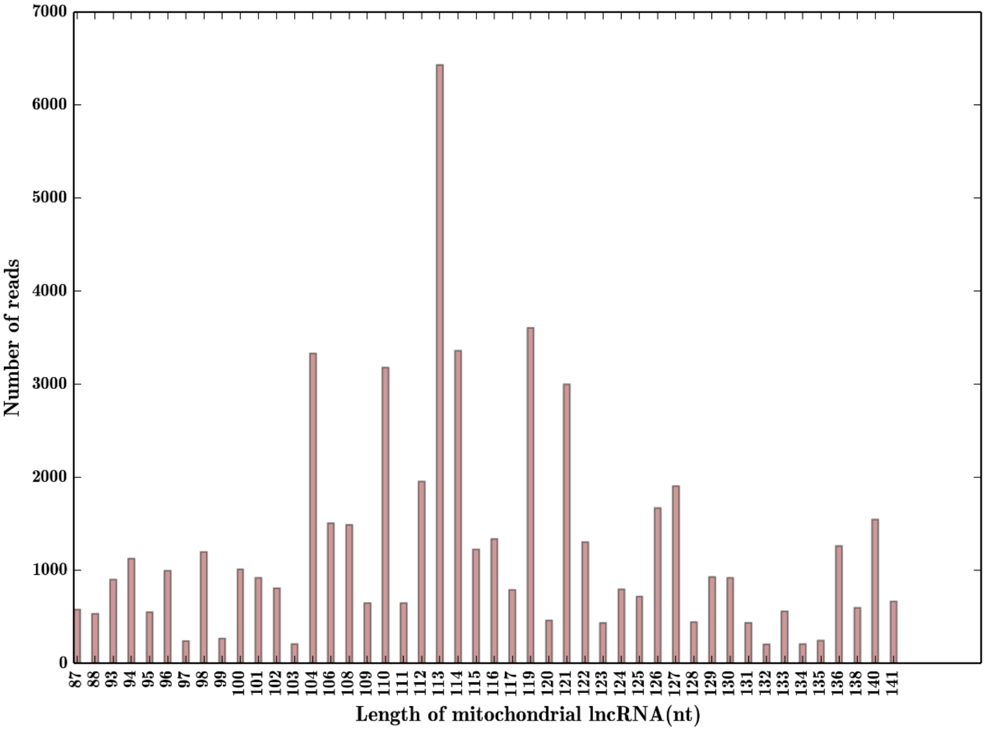
Number of reads
Length of mitochondrial lncRNA(nt)
0
1000
2000
3000
4000
5000
6000
7000
87 88 93 94 95 96 97 98 99 100 101 102 103 104 106 108 109 110 111 112 113 114 115 116 117 119 120 121 122 123 124 125 126 127 128 129 130 131 132 133 134 135 136 138 140 141

Figure S5A

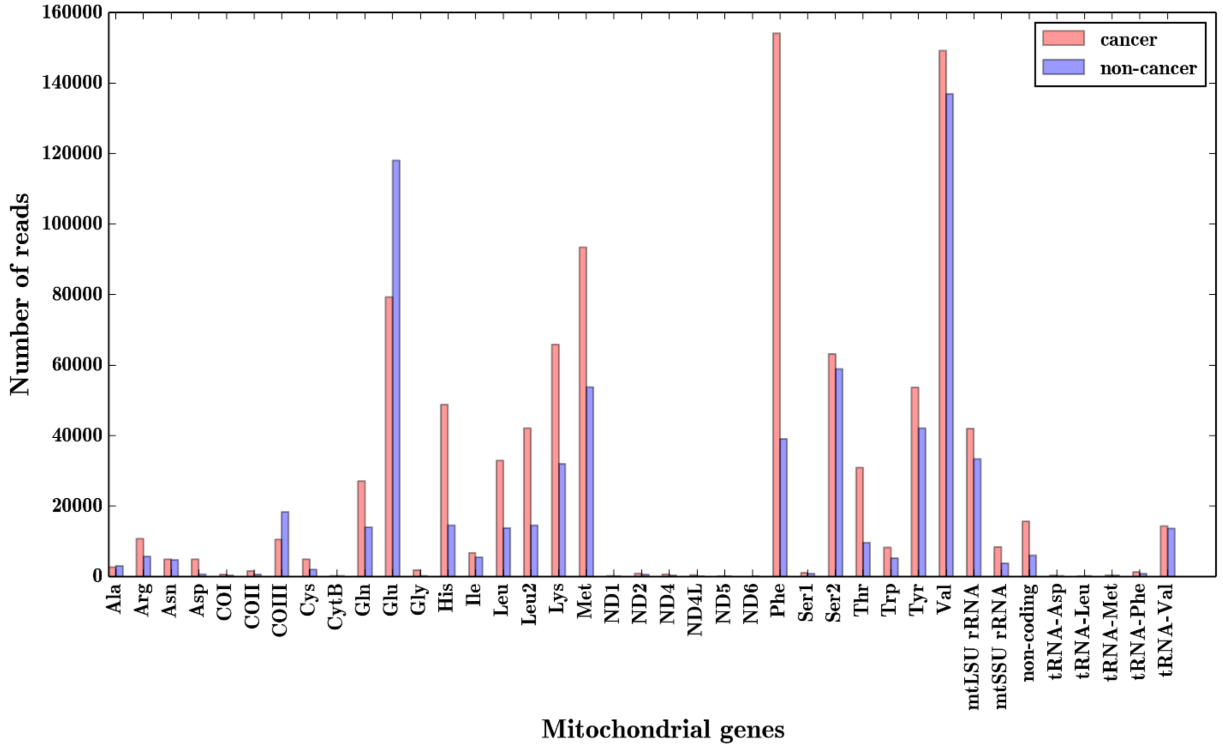

Figure S5B

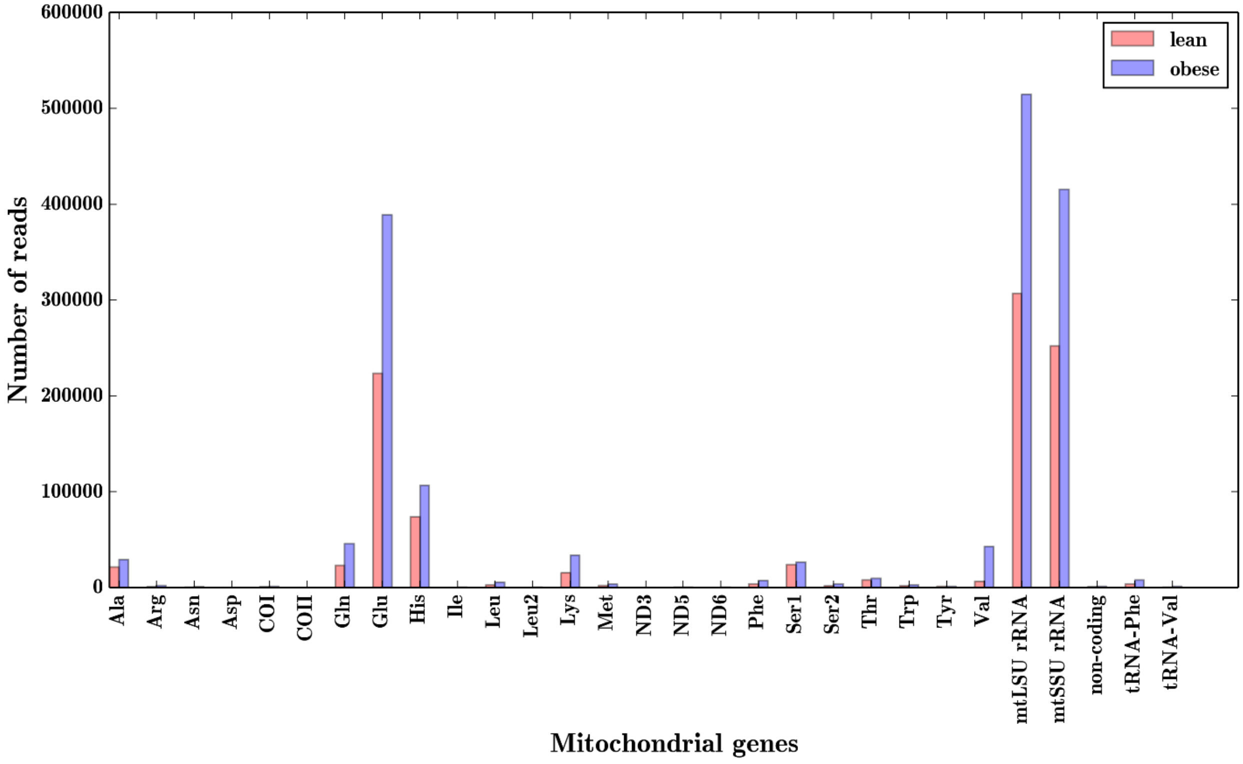

Figure S5C

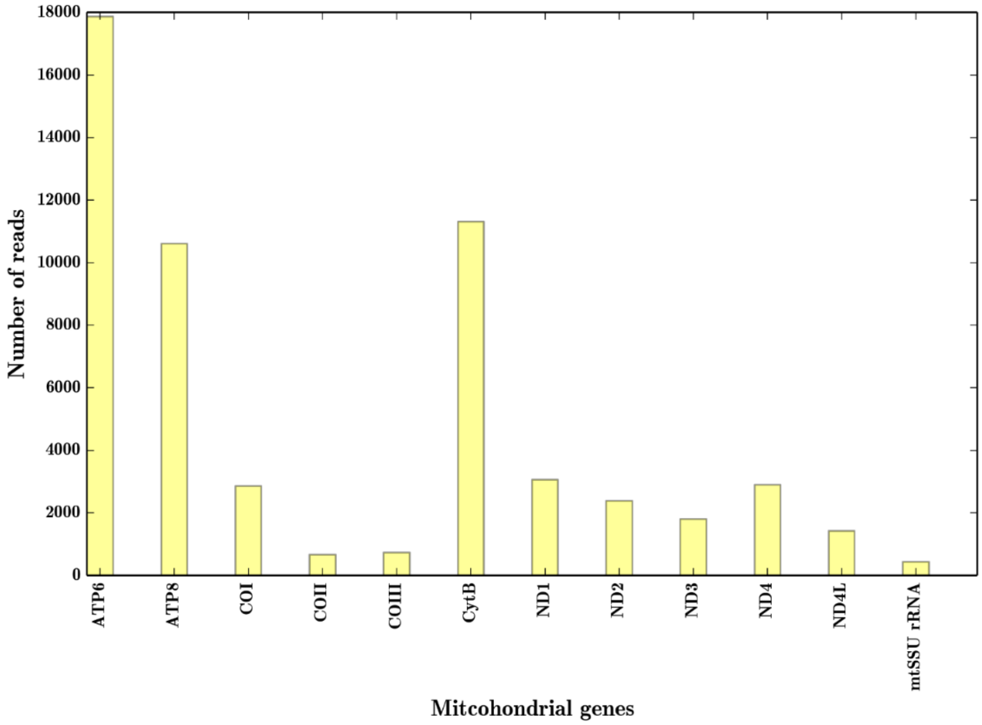

18000
16000
14000
12000
10000
8000
6000
4000
2000
0
Number of reads
ATP6
ATP8
COI
COII
COIII
CytB
ND1
ND2
ND3
ND4
ND4L
mtSSU rRNA
Mitcohondrial genes

Figure S6

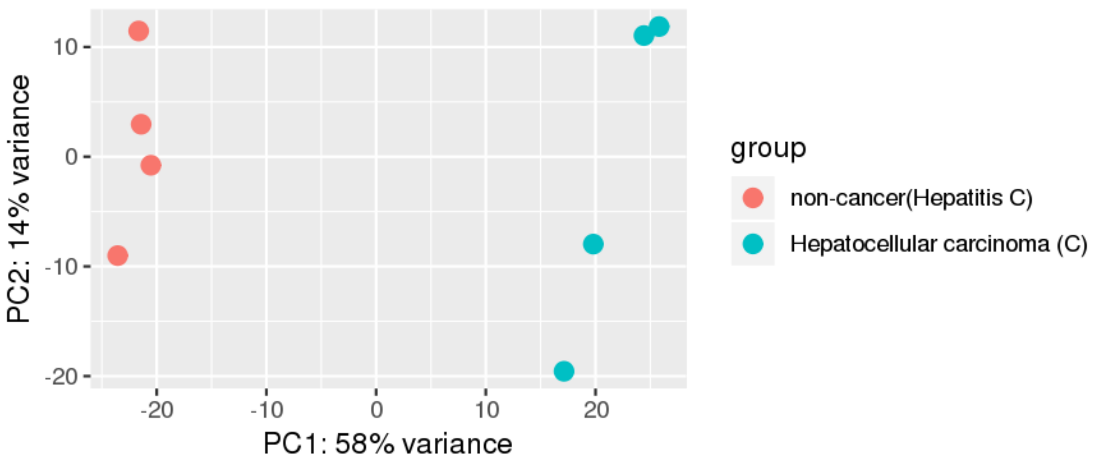

Figure S7

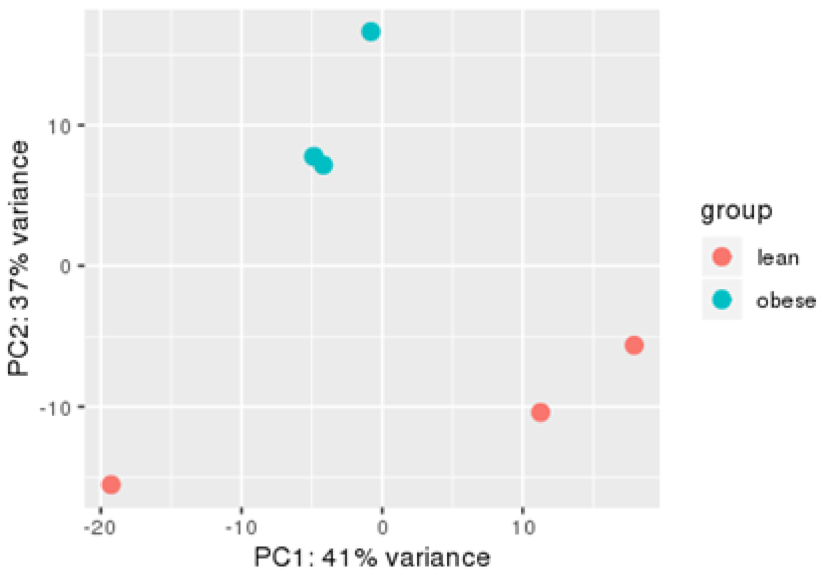

Supplement: Supplementary file 1 [file ijms-24-04373-s001.zip › Supplementary File S8.pdf]
